# Induced in-source fragmentation pattern of certain novel (*1Z,2E*)-*N*-(aryl)propanehydrazonoyl chlorides by electrospray mass spectrometry (ESI-MS/MS)

**DOI:** 10.1186/1752-153X-7-16

**Published:** 2013-01-25

**Authors:** Ali S Abdelhameed, Mohamed W Attwa, Hatem A Abdel-Aziz, Adnan A Kadi

**Affiliations:** 1 Department of Pharmaceutical Chemistry, College of Pharmacy, King Saud University, P.O. Box 2457, Riyadh 11451, Kingdom of Saudi Arabia

**Keywords:** In-source fragmentation, Hydrazones, Fragmentor voltage, Electrospray ionization

## Abstract

**Background:**

Collision induced dissociation (CID) in the triple quadrupole mass spectrometer system (QQQ) typically yields more abundant fragment ions than those produced with resonance excitation in the presence of helium gas in the ion trap mass spectrometer system (IT). Detailed product ion spectra can be obtained from one stage MS^2^ scan using the QQQ. In contrast, generating the same number of fragment ions in the ion trap requires multiple stages of fragmentation (MS^n^) using CID via in-trap resonance excitation with the associated time penalties and drop in sensitivity.

**Results:**

The use of in-source fragmentation with electrospray ionization (ESI) followed by product ion scan (MS^2^) in a triple quadrupole mass spectrometer system, was demonstrated. This process enhances the qualitative power of tandem mass spectrometry to simulate the MS^3^ of ion trap for a comprehensive study of fragmentation mechanisms. A five pharmacologically significant (*1Z*, *2E*)-*N*-arylpropanehydrazonoyl chlorides **(3a-e)** were chosen as model compounds for this study. In this work, detailed fragmentation pathways were elucidated by further dissociation of each fragment ion in the ion spectrum, essentially, by incorporating fragmentor voltage induced dissociation (in-source fragmentation) and isolation of fragments in a quadrupole cell Q1. Subsequently, CID occurs in cell, Q2, and fragment ions are analyzed in Q3 operated in product ion mode this process can be referred to as pseudo-MS^3^ scan mode.

**Conclusions:**

This approach allowed unambiguous assignment of all fragment ions using tandem mass spectrometer and provided adequate sensitivity and selectivity. It is beneficial for structure determination of unknown trace components. The data presented in this paper provide useful information on the effect of different substituents on the ionization/fragmentation processes and can be used in the characterization of this important class of compounds.

## Background

Several types of ionization methods are available for use in mass spectrometry studies, such as electrospray ionization (ESI), matrix assisted laser desorption ionization (MALDI), chemical ionization (CI), atmospheric pressure chemical ionization (APCI), or electron impact (EI). Electrospray ionization mass spectrometry (ESI-MS), as a powerful tool for the analysis, has been widely applied to the analysis of glycoproteins [1], oligonucleotides [2,3], oligosaccharides [4,5], drugs and drug metabolites [6], environmental contaminants [7,8], and numerous other types of compounds [9]. In atmospheric pressure ion sources, e.g. APCI or electrospray ionisation (ESI), fragmentation of ions can occur in the ion source (in-source fragmentation) before ions reach the analyzer. In spite of the fact that this method of in source fragmentation is less selective than tandem mass spectrometry, as all ions in the source will be fragmented simultaneously, it has been used by several research groups [10-19]. On the other hand, collision induced dissociation (CID) in the triple quadrupole mass spectrometer system (QQQ) typically yields more abundant fragment ions than those produced with resonance excitation in the presence of helium gas in the ion trap (IT) [20]. Detailed product ion spectra can be obtained from one stage MS^2^ scan using the QQQ. In contrast, generating the same number of fragment ions in the ion trap requires multiple stages of fragmentation (MS^n^) using CID via in-trap resonance excitation with the associated time penalties and drop in sensitivity. Another drawback to the IT is the low mass cut-off in product ion scans. This arises from the fact that the precursor ion and the lowest *m*/*z* fragment ion must be stable simultaneously in the IT [20,21]. In the present study, we combine the usage of in-source fragmentation with product ion scan (MS^2^) to obtain structural information of certain novel (*1Z2E*)-*N*-(aryl)propanehydrazonoyl chlorides using electrospray ionization tandem mass spectrometry (ESI-MS/MS). The use of this approach was designed to improve the structure elucidation power of tandem mass spectrometry to simulate the MS^3^ of ion trap overcoming the aforementioned drawbacks of the latter.

**Scheme 1:**
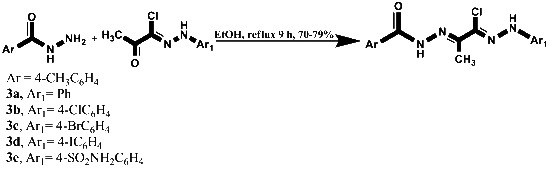
Synthesis of compounds 3a–e.

Hydrazones have been reported as useful antiviral agents. Some acetylhydrazones revealed a remarkable antiviral activity against HSV-1 [22], whereas arylhydrazones inhibited the replication of HIV-1 [23]. Recently, Abdel-Aziz *et al*. reported the synthesis of a new class of (1*Z*,2*E*)-*N*-(aryl)propanehydrazonoyl chlorides as analogs of compounds **3a-e** (Scheme 1) [24,25] the structure and absolute configuration of this new class of hydrazonoyl chlorides were confirmed using X-ray analyses [25]. Furthermore, these derivatives were also used in economical and versatile synthetic approach for stereo-selective synthesis of novel amidrazone derivatives with significant antifungal [25], and antiviral [24] potencies. Additionally, they can be considered very promising in the perspective of new drugs discovery. Amidrazones were also found to possess interesting biological activities [26-29]. Some of amidrazone-containing hetererocycles have demonstrated potent activity in vitro as antimicrobial agents. Amidrazone-substructural fragments were found to be the antifungal pharmacophore of the latter compounds [30-32]. Amidrazones have also been reported as precursors of some effective antifungal azoles [33]. Consequently, the efficient construction of these molecules has received significant attention [34-38]. Accordingly, in the light of interesting structural and biological results the situation definitely urges for some additional research in their analyses.

## Results and discussion

### Chemistry

General procedure for the synthesis of (*1Z*,*2E*)-2- (benzoyl/benzothiazol-2-oyl)-N-arylpropanehydrazonoyl chlorides **3a–e**. A mixture of benzoyl hydrazine 1a or benzothiazole-2-carbohydrazide 1b (10 mmol) and 2-oxo-N-arylpropanehydrazonoyl chloride **2a–d** (10 mmol) in absolute ethanol (50 mL) was refluxed for 9 h. Then left to cool, the formed solid was filtered off, washed with ethanol, and recrystallized from EtOH/DMF to afford the corresponding hydrazonoyl chlorides **3a–e**, respectively. The target compounds were synthesized via reacting 4-methylbenzohydrazide (**1**) with 2-oxo-*N*-arylpropanehydrazonoyl chlorides **2a-e** in refluxing ethanol. The latter reaction produced, in each case, a single yellow product was identified as (1*Z*,2*E*)-2-(2-(4-methylbenzoyl)hydrazono)-*N*’-arylpropanehydrazonoyl chloride **3a-e**.

### Mass spectrometry

**Figure 1:**
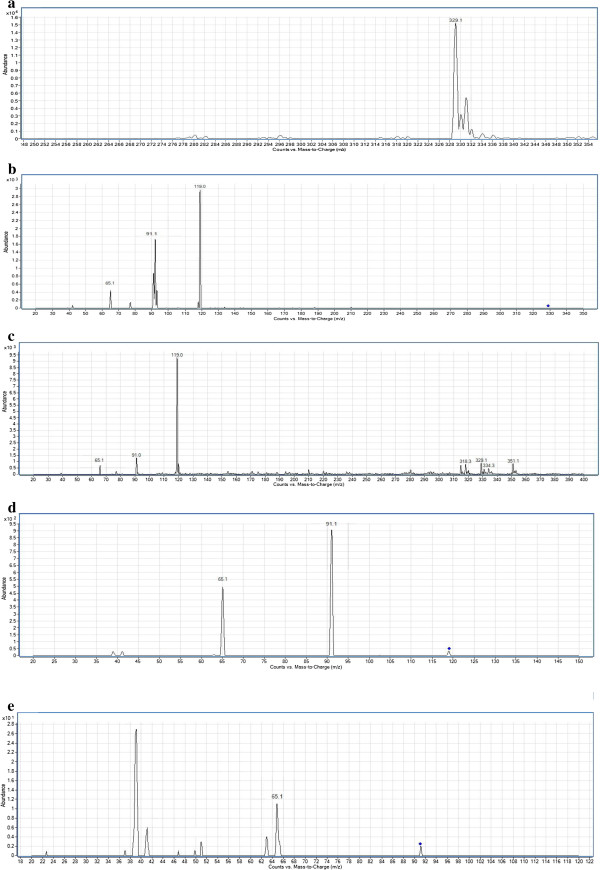
**a) ESI mass spectrum of compound 3a [M+H]**^**+**^**ion (m/z 328.80). b**) MS^2^ spectrum of m/z 328.80. **c**) In-source fragmentation of compound **3a. d**) MS^2^ spectrum of m/z 119.14. **e**) MS^2^ spectrum of m/z 91.13.

**Figure 2:**
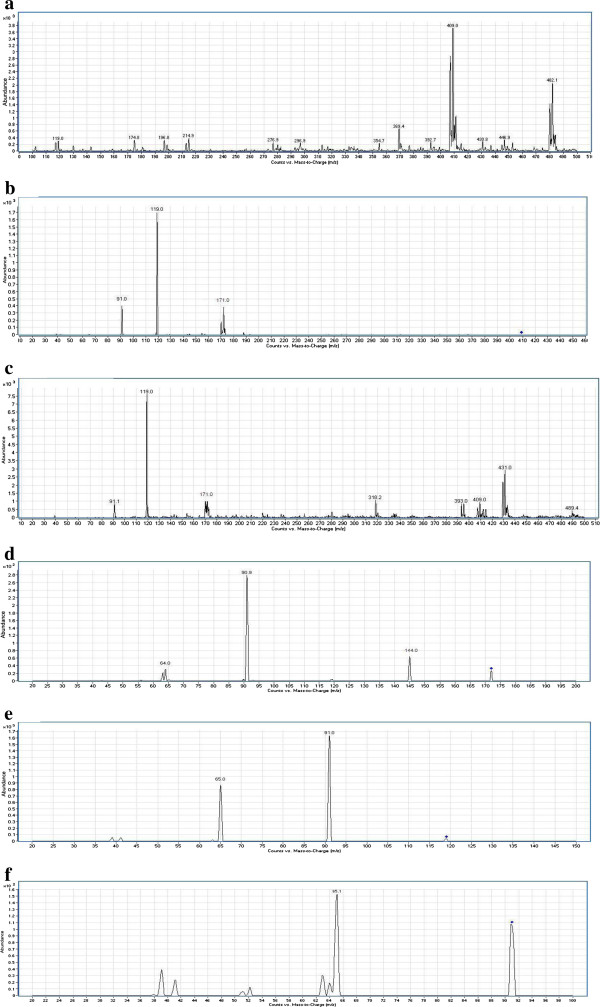
**a) ESI mass spectrum of compound 3c [M+H]**^**+**^**ion (m/z 409). b)** MS^2^ spectrum of m/z 409. **c)** In-source fragmentation of compound **3c. d)** MS^2^ spectrum of m/z 171.01. **e)** MS^2^ spectrum of m/z 119.14. **f)** MS^2^ spectrum of m/z 91.13.

A preliminary MS2 scan followed by a product ion scan of each compound was carried out to determine the parent ion peaks as well as the fragment ions of compounds **3a-e**. The data obtained played a guidance role prior to the pseudo-MS^3^ process for the same compounds. The highly sensitive product ion spectra of compounds **3a-e** obtained from a single-stage MS^2^ scan with abundant product ions and no low mass cut-off, are represented in Figures 1 and 2.

**Scheme 2:**
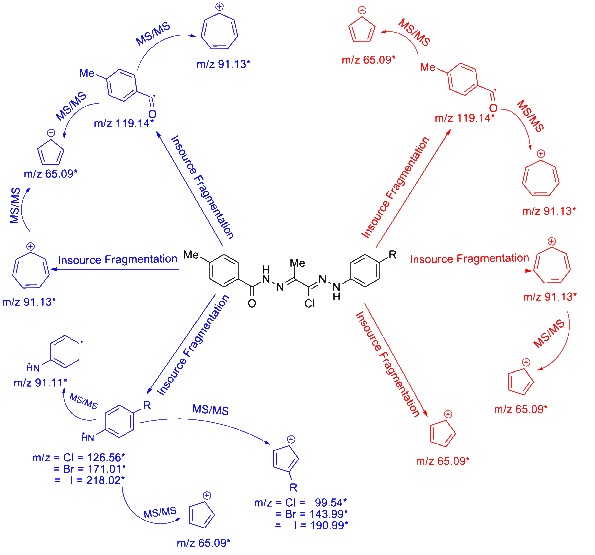
**Proposed fragmentation pattern of compounds 3a-e conducted with Pseudo-MS**^**3**^**scan mode in ESI-MS/MS.**

The fragmentation pathways of compounds **3a-e** were investigated (Scheme 1). Introduction of different substituents to the investigated compounds showed marked effect on their ionization/fragmentation pattern. In-source fragmentation of (*1Z*, *2E*)-*N*-arylpropanehydrazonoyl chlorides that have an electron donating substituent on ring B (X= H or SO_2_NH_2_) showed limited fragmentation at the N/-C=O and Ar/-C=O bonds, with increased stability of the substituted ring B. This fragmentation mechanism produced three ion peaks with m/z 65.09, 91.13 and 119.14, MS^2^ scan of the last two fragment ions produced m/z 65.09 and 91.13 with 65.09 respectively. Whereas, presence of an electron withdrawing substituent (Cl, Br or I) on ring B, produces similar fragmention mechanism in addition to yielding another pattern with dissociation occurs at the N-N bond linked to the substituted ring B producing a protonated fragment with m/z 126.56 or 171.01 or 218.02 for Cl or Br or I, respectively. MS^2^ scan of this fragment ion shows three ion peaks m/z 91.13, 65.09 and 99.54 or 143.99 or 190.99 for Cl or Br or I, respectively. Ion peaks together with their corresponding proposed structures obtained from in-source fragmentation and MS^2^ scans for compound **3a-e** are shown in Scheme 2 and are also summarized in Table 1.

**Table 1:** Multistage MS data of compounds 3a-e by ESI-MS/MS

**Product ions of protonated compounds 3a-e [M+H]**^**+**^**(m/z)**					
	**3a (328.80)**	**3b (363.24)**	**3c (409.0)**	**3d (454.69)**	**3e (407.87)**
In-source fragmentation	65.09				65.09
	91.13	91.13	91.13	91.13	91.13
	119.14	119.14	119.14	119.14	119.14
		126.56			
			171.0		
				218.02	
Product ion	65.09	65.09	65.09	65.09	65.09
	91.13	91.13	91.13	91.13	91.13
		99.54			
			143.99		
				190.99	

## Experimental

### General method for synthesis of (1Z,2E)-2-(2-(4-methylbenzoyl)hydrazono)-N’-(aryl)propanehydrazonoyl chloride 3a-e

**Figure 3:**
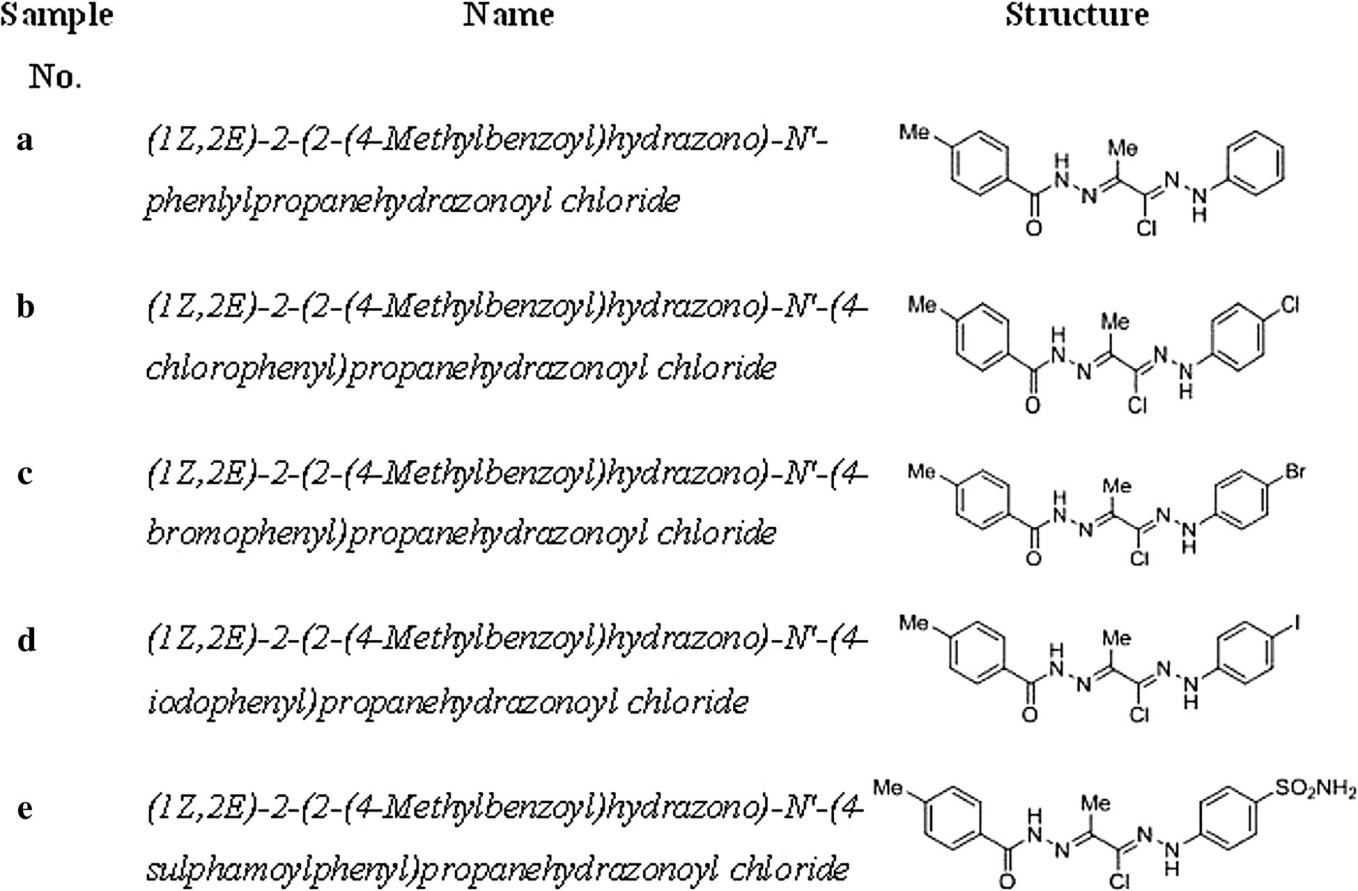
**Structure of the (1** ***Z*,2** ***E*)-*N*-arylpropanehydrazonoyl chlorides 3a-e.**

Unless otherwise indicated, all chemicals were purchased from Sigma (St. Louis, MO). To a solution of hydrazide **1** (0.15 g, 1 mmol) in ethanol (30 mL), the appropriate propanehydrazonoyl chloride **2** (1 mmol) was added. The reaction mixture was heated under refluxing temperature for 6 h, then left to cool at room temperature. The precipitated product was filtered off, washed with ethanol and recrystallized from EtOH/DMF to produce the corresponding (1*Z*,2*E*)-*N*-arylpropanehydrazonoyl chlorides **3a-e** in 75–85% yield. The structures of all new compounds are shown in Figure 3.

## Mass spectrometry

### Reagents and solvents

HPLC grade water was obtained by Milli-Q connected to Elix Millipore water purification system (Millipore, USA). Acetonitrile (ACN) HPLC grade was purchased from BDH laboratory supplies (Poole-UK).

### LC-MS/MS

An Agilent 6410 triple quadrupole mass spectrometer (Agilent technologies, USA) equipped with an electrospray ionization interface (ESI) coupled to an Agilent 1200 HPLC (Agilent Technologies, USA) was used. Agilent 1200 series system consists of G1311A binary pump, G1322A degasser, G1367B HIP-ALS autosampler, and G1316A thermostatted column compartment. A connector is used instead of the column to allow direct injection of samples. Mobile phase composed of two solvents: A is HPLC grade water, and B is acetonitrile mixed in the ratio 1:1. Compounds were prepared by weighing the solid substances to 1 mg.mL^-1^ in ACN. Test solutions for MS were prepared by diluting the stock solutions with ACN/H2O mixture (1:1). Flow rate is 0.4 ml/min, run time was 3 minutes. 10 μl of each sample was injected into the LC-MS/MS. MS parameters were optimized for each compound by varying fragmentor voltage of the ion source for scan mode and collision energy for product ion mode. For optimization of the ionization conditions and of fragment ion spectra, analytes concentrations of 20 to 50 μg.mL^-1^-depending on the ions intensities- were employed. For screening of mass signals of the different compounds and to search for parent ions for MS/MS experiments, MS2 scans were performed in the mass range of m/z 100–600. Because of the flow rate dependency of the ESI process, ion source specific parameters were readjusted. The ESI was operated in positive mode. The source temperature was set to 350°C and ion spray voltage was 4.5 kV.

To overcome the reduced selectivity problem of in-source fragmentation, a product ion scan was performed prior to the in-source fragmentation to determine each compound’s related fragment ions. The fragmentor voltage was optimized to produce adequate in-source fragmentation; values of 100, 120,140,160,180 and 200 V were tested to obtain the fragments of each compound in the scan spectra. The optimum fragmentor voltage to generate in-source fragments was 200 V. Furthermore, the collision energy used for product ion (MS^2^) analysis was also optimized by varying collision energy values (4, 6, 8, 10, 12, 14, 16, 18 and 20 eV) and was set to 20 eV to attain the fragment ions.

## Conclusions

Mass spectrometry has shown to be an effective tool for the structural characterization of the synthesized compounds **3a-e**. This work demonstrated a comprehensive fragmentation mechanism study of compounds **3a-e** using the combination of induced in-source fragmentation with product ion scan (MS^2^). The highly sensitive product ion spectra of compounds **3a-e** were obtained from a single-stage MS^2^ scan with abundant product ions and no low mass cut-off. The detailed fragmentation pathways of all ions observed in the in-source fragmentation spectrum of compounds **3a-e** were elucidated by further dissociation of each of these fragment ions using the product ion (MS^2^) scan mode. The substructures of all fragment ions were unambiguously assigned. The approach described here is simple, sensitive, rapid and powerful for the identification of unknown compounds. Additionally, it can be used to identify trace amounts of metabolites and can be potentially applied to the analysis of impurities and degradation products in complex systems.

## Abbreviations

CID: Collision induced dissociation; QQQ: Triple quadrupole mass spectrometer system; IT: Ion trap mass spectrometer system; MS^n^: Multiple stages of fragmentation; ESI: Electrospray ionization; MALDI: Matrix assisted laser desorption ionization; APCI: Atmospheric pressure chemical ionization; CI: Chemical ionization; ESI-MS: Electrospray ionization mass spectrometry; EI: Electron impact; ACN: Acetonitrile; HPLC: High-performance liquid chromatography.

## Competing interests

The authors declare that they have no conflict of interests.

## Authors’ contributions

ASA proposed the subject, designed the study, and wrote the draft version of the manuscript. MA conducted the mass spectrometric analysis of the compounds. HA conducted the synthesis of the investigated compounds and wrote the chemistry part of the manuscript. AK participated in study design, literature review, mass spectrometric analysis and preparation of the manuscript. All authors read and approved the final manuscript.
